# Soluble Beta-Amyloid Peptides, but Not Insoluble Fibrils, Have Specific Effect on Neuronal MicroRNA Expression

**DOI:** 10.1371/journal.pone.0090770

**Published:** 2014-03-04

**Authors:** Jing Jing Li, Georgia Dolios, Rong Wang, Francesca-Fang Liao

**Affiliations:** 1 Department of Pharmacology, University of Tennessee Health Science Center, Memphis, Tennessee, United States of America; 2 Department of Genetics and Genomic Sciences, Mount Sinai School of Medicine, New York, New York, United States of America; University of the Witwatersrand, South Africa

## Abstract

Recent studies indicate that soluble β-amyloid (sAβ) oligomers, rather than their fibrillar aggregates, contribute to the pathogenesis of Alzheimer's disease (AD), though the mechanisms of their neurotoxicity are still elusive. Here, we demonstrate that sAβ derived from 7PA2 cells exert a much stronger effect on the regulation of a set of functionally validated microRNAs (miRNAs) in primary cultured neurons than the synthetic insoluble Aβ fibrils (fAβ). Synthetic sAβ peptides at a higher concentration present comparable effect on these miRNAs in our neuronal model. Further, the sAβ-induced miR-134, miR-145 and miR-210 expressions are fully reversed by two selective N-methyl-d-aspartate (NMDA) receptor inhibitors, but are neither reversed by insulin nor by forskolin, suggesting an NMDA receptor-dependent, rather than PI3K/AKT or PKA/CREB signaling dependent regulatory mechanism. In addition, the repression of miR-107 expression by the sAβ containing 7PA2 CM is likely involved multiple mechanisms and multiple players including NMDA receptor, N-terminally truncated Aβ and reactive oxygen species (ROS).

## Introduction

Alzheimer's disease (AD) is pathologically characterized by extracellular amyloid plaques and cytoplasmic tau tangles, which are believed to contribute to neurodegeneration (synapse loss and cell death) and cognitive impairment [1]. The insoluble amyloid β fibrils (fAβ) which constitute the extracellular plaques were used to be considered a major pathogenic factor in AD for two decades [2]. However, overwhelming new evidence supports soluble Aβ (sAβ) oligomers as an early trigger of synaptic damage and cognitive impairment in AD. These include the weak correlation between the fAβ and synaptic loss, neuronal death, or cognitive impairment [3], [4], [5], the strong correlation between sAβ levels and the severity of neuropathological changes in AD, as well as the potent ability of sAβ to cause synaptic failure and cognitive function disruption [6], [7].

The prefibrillar sAβ are found to be more toxic than their insoluble fibrillar counterparts. Exposure of hippocampal neurons to synthetic Aβ [8] or to cell-derived sAβ [9] induce progressive synaptic loss. The sAβ extracted directly from AD brains inhibit long-term potentiation (LTP), enhance long-term depression (LTD), and reduce dendritic spine numbers when injected into rodent brains [10]. Recently, sAβ have been reported to induce marked neuronal loss and disrupt hippocampus-dependent memory when injected into awake, freely moving mice [11]. The exact mechanisms underlying how sAβ lead to neuronal dysfunction remain only partially understood.

miRNAs, whose sequences are highly conserved across eukaryotic species, are short non-coding RNA molecules (∼22 nucleotides). In recent years, many studies have highlighted the importance of miRNAs as a powerful class of gene regulators in various biological processes. Using microarray analysis or northern blot hybridization, the particular expression profiles of many brain-expressed miRNAs that are associated with normal brain development and neuronal differentiation have been identified [12], [13], [14], [15]. Most interestingly, some miRNAs are found to be regulated by neuronal activity [16], [17], [18], control synaptic plasticity [19], [20], [21], [22], or even participate in the formation of memory [23], [24], [25]. On the other hand, increasing evidence suggests that dysregulated miRNAs contribute directly in the pathogenesis of a variety of human diseases, including neurodegenerative diseases [26]. A number of miRNA expression patterns are found to be altered in AD patients' brains [27], [28], [29], [30], [31], [32], [33], [34] and in the brains of AD mouse models [35], [36], [37], [38]. However, the cause of their deregulation and how their deregulation affects AD progression are mostly unknown. We hypothesize that the pathogenic sAβ are able to alter the expressions of a specific set of miRNAs that are deregulated in AD brains.

Given that the biological outcomes resulting from distinct assemblies of Aβ species are different, the Aβ-mediated mechanisms of AD progression may thus differ by different Aβ species. The aim of this study was to test whether sAβ and fAβ differentially regulate the expression of a subset of 9 miRNAs that was previously reported to be aberrantly expressed in AD or was well-demonstrated in the regulation of synaptic plasticity, inflammation, apoptosis, or mitochondrial activity. In this study, we treated mature primary cortical neurons with soluble human Aβ naturally derived from the conditioned medium of 7PA2 cells, which contains a combination of monomers, dimers, trimers and other oligomers, as opposed to the fAβ prepared by using synthetic Aβ_1–42_ or Aβ_25–35_ peptides, and determined expressional alterations of these selected miRNAs by quantitative real-time PCR (qRT-PCR).

## Materials and Methods

### Ethic statement

All animal work was approved by the Institutional Animal Care and Use Committee (IACUC) of the University of Tennessee Health Science Center (UTHSC).

### Primary neuron cultures

Primary cortical neurons were isolated from E17 embryos of Sprague Dawley rats as described previously [39]. All experiments presented in this work were performed on mature neuronal cells at 14 days in vitro (DIV) except as otherwise noted.

### Chemicals and antibodies

DL-2-amino-5-phosphonopentanoic acid (AP5), ifenprodil, 30% hydrogen peroxide (H_2_O_2_), piceid, forskolin, Hexafluoro-2-propanol (HFIP), glutaraldehyde, Trichloroacetic Acid (TCA) and human recombinant insulin were purchased from Sigma (St Louis, MO). Anti-MAP2 was from Sigma (St Louis, MO) Anti-APP (1G6) mouse monoclonal antibody raised against amino acid 573-596 of APP was purchased from Axxora; anti-APP (22C11) mouse monoclonal antibody against N terminus APP was developed in our laboratory; anti-Aβ (N terminus amino acid 1-12) mouse monoclonal antibody B436 was a gift from Dr. Steve L. Wagner (TorreyPines Therapeutics, Inc.); anti-Aβ (amino acid residues 17–24) mouse monoclonal antibody 4G8 was purchased from Covance. Anti-phospho-IGF-I Receptor β (Tyr1135/1136)/Insulin Receptor β (Tyr1150/1151) (19H7), anti-IRβ, anti-phospho-CREB (Ser133), and anti-CREB antibodies were from Cell Signaling Technology (Danvers, MA).

### Naturally secreted Aβ-containing CM, control medium, and Aβ peptides preparation

The 7PA2 cells are Chinese Hamster Ovary (CHO) cells stably transfected with human APP_751_ which contains a Val717Phe mutation. Medium containing soluble human Aβ was derived from the conditioned medium of 7PA2 cells. Briefly, 7PA2 cells were grown in Dulbecco's modified Eagle's medium (DMEM, HyClone) containing 10% characterized fetal bovine serum (FBS, HyClone) and 200 µg/ml G418 (Calbiochem). Cells were washed with sterilized PBS at 80–90% confluence and conditioned in 5 ml of B27-free neurobasal medium (Invitrogen, Carlsbad, CA) for ∼16 hr. Afterwards, 7PA2 CM was removed and cleared of cells by passage through a sterile 0.22 µM filter (EMD Millipore, Billerica, MA). The control medium was derived likewise from CHO cells cultured in DMEM containing 10% FBS. Aliquots of 7PA2 CM and CHO CM were stored at −80°C Before use; the CM was supplemented with B27 and glutamine. A 1∶1 dilution of the CM was used to treat neurons. Synthetic human Aβ_25–35_ and Aβ_1–42_ peptides were purchased from AnaSpec (Fremont, CA). The lyophilized Aβ_25–35_ peptide was dissolved in sterilized water (pH7.4) to a final concentration of 250 µM. Soluble oligomeric Aβ_25–35_ was prepared by incubating the Aβ solution at 4°C for 24 hr. The fibrillar Aβ_25–35_ was prepared by incubating at 37°C for 24 hr and then spinning at 14,000 *g* for 10 min to sediment the insoluble fibrils [40]. The protein concentration in the supernatant was determined by a BCA protein assay kit (Thermo Fisher Scientific, Waltham, MA) to confirm that over 90% of the Aβ peptide were fibrilized and precipitated. Fibrils were resuspended in water by vigorous vortexing prior to pipetting aliquots for cell stimulation. The synthetic Aβ_1–42_ peptide was first suspended to an initial concentration of 1 mM in HFIP followed by incubation for 2 hr at room temperature. The solvent was then evaporated in a Savant SpeedVac Concentrator with an Ultra-low temperature refrigerated vapor trap. Peptide was subsequently re-suspended in dry DMSO as monomers and frozen at −80°C until use. Oligomerization and fibrillation procedures of the Aβ_1–42_ were similar to those of the Aβ_25–35_.

### Immunodepletion

Briefly, 3 µg of antibody and 30 µl of protein A/G beads (Thermo Fisher Scientific, Waltham, MA) were added to 1 mL of 7PA2 CM for 8 hr at 4°C. Three cycles of immunoprecipitation were performed to ensure complete removal of antigens from 7PA2 CM.

### SDS-PAGE and immunoblotting on Aβ species

Different Aβ preparations were re-suspended in 10 µL of 2X Novex Tricine SDS sample buffer (Invitrogen, Carlsbad, CA) and boiled in water for 3 min. For Western blots, samples were electrophoresed on a Novex 10–20% Tricine gel with 1X Novex Tricine SDS Running Buffer (Invitrogen, Carlsbad, CA). Proteins were transferred onto a 0.2 µm PVDF membrane and the membrane was briefly fixed with 0.2% glutaraldehyde at room temperature (RT) for 30 min, blocked at RT in 1X Tris Buffered Saline plus 0.05% Tween-20 (TBST) containing 5% non-fat dry milk for 1 hr, and incubated overnight at 4°C in primary antibody (B436; 1∶1000 in TBST/5% BSA/0.02% NaN_3_). On the second day, the membrane was washed three times with TBST and then incubated in secondary horseradish peroxidase (HRP) linked anti-mouse antibody (GE Healthcare Life Sciences, 1∶5000 in blocking buffer). Blot was washed with TBST and applied with enhanced chemiluminescence (ECL) for signal development. For non-Aβ-detection samples, Tris-Glycine gel and buffer system were applied.

### Immunofluorescence staining

Primary cortical neurons seeded on coverslips were fixed with 4% paraformaldehyde prepared in PBS at RT for 15 min. After brief washing, neurons were blocked and permeabilized in PBS containing 5% goat serum and 0.1% Triton X-100 at room temperature for 1 hr in a humid chamber. Anti-MAP2 antibody (1∶500, Sigma) was applied to the coverslips and incubated overnight at 4°C. After extensive washes, Alexa 488-conjugated anti-mouse antibody (1∶500, Invitrogen) was applied and incubated for 1 hr at RT. Slides were mounted with Fluoromount medium (Sigma, St Louis, MO) prior to image capturing under a Leica microscope. For immunostaining of the intracellular reactive oxygen species (ROS), neurons on coverslip were incubated with 10 µM of 5-(and 6-)chloromethyl-2′,7′-dichlorodihydrofluorescein diacetate, acetyl ester (CM-H2DCFDA) (Invitrogen, Carlsbad, CA) in sterilized PBS for 30 min in the dark. After labeling, CM-H2DCFDA was removed from neurons by washing with sterilized PBS and neurons replenished with culture medium. After treatments, cells were fixed with 4% paraformaldehyde in the dark. Production of ROS was measured by fluorescence microscopy.

### RNA isolation, cDNA synthesis, and qRT-PCR

Total RNA was isolated using Trizol reagent (Invitrogen, Carlsbad, CA). Briefly, cells cultured on a 100-mm dish were lysed by applying 1 ml of Trizol reagent. Samples were segregated into phenol-chloroform phases. The aqueous supernatant phase was transferred to an RNase-free tube and precipitated with isopropanol. The RNA pellet was washed twice with 70% ethanol prepared with DEPC-treated water, air dried, and dissolved in RNase-free water (Thermo Fisher Scientific, Waltham, MA). The cDNAs were synthesized from the prepared total RNA using NCode miRNA First Strand cDNA synthesis kit (Invitrogen, Carlsbad, CA) according to the manufacture's protocol. The amount of miRNA was detected with 5 Prime RealMasterMix SYBR ROX (5 Prime) and an Eppendorf Mastercycler realplex Real-Time PCR system. The quantitative real-time PCR runs were performed under the following thermocycler conditions: initial denaturation at 95°C for 2 min, followed by 40 cycles of 95°C for 15 s, 55°C for 15 s, and 68°C for 20 s. Primers for mature miRNAs designed using their rat sequences from miRBase are listed in Table 1. The expression of miRNAs was normalized to 5S rRNA. 5S primers: forward 5′-GCCCGATCTCGTCTGATCT-3′; reverse 5′-GCCTACAGCACCCGGTATC-3′. U6 primers: forward 5′- GTGCCTGCTTCGGCAGCAC -3′; reverse 5′- GTGTCATCCTTGCGCAGGG-3′.

**Table 1:** List of primer sequences used for miRNA detection and the identified properties of tested miRNAs.

_miRNA_	_Accession Number_	_Primers Sequence_	_Identified Properties_
_miR-107_	_MIMAT0000826_	_AGCAGCATTGTACAGGGCTATCA_	_P53 induced [74]; deceased early in AD [27]_
_miR-124_	_MIMAT0000828_	_TAAGGCACGCGGTGAATGCC_	_Promote neural differentiation; synaptic plasticity [20]_
_miR-125b_	_MIMAT0000830_	_TCCCTGAGACCCTAACTTGTGA_	_Promote neural differentiation; synaptic plasticity [21]_
_miR-132_	_MIMAT0000838_	_TAACAGTCTACAGCCATGGTCG_	_Neuron activity dependent; CREB-regulated [21, 22]_
_miR-134_	_MIMAT0000840_	_TGTGACTGGTTGACCAGAGGGG_	_Mef2 induced; neuron activity dependent [19]_
_miR-145_	_MIMAT0000851_	_GTCCAGTTTTCCCAGGAATCCCT_	_P53 induced; pro-apoptotic [57, 58]_
_miR-146a_	_MIMAT0000852_	_TGAGAACTGAATTCCATGGGTT_	_NF-kappaB-dependent; inflammation-associated [56]_
_miR-210_	_MIMAT0000881_	_CTGTGCGTGTGACAGCGGCTGA_	_Hypoxia induced; HIF-1a dependent [66]_
_miR-338_	_MIMAT0000581_	_TCCAGCATCAGTGATTTTGTTG_	_Inhibit mitochondrial activity [75]_

### Immunoprecipitation mass spectrometry (IPMS)

IPMS measurement of Aβ peptides was carried out as described previously [41] except the antibodies indicated. Aβ peptides in 7PA2 CM (6 mL) were immunoprecipitated by incubating overnight with antibody 6E10 or B436 and Protein A/Protein G plus beads. Mass spectra were collected using a TOF/TOF 5800 mass spectrometer (AB Sciex). Each mass spectrum was accumulated from 2,000 laser shots and calibrated using bovine insulin (an internal mass calibrant).

### Statistical analysis

GraphPad Prism 5 software was used to perform data analysis. All data are presented as mean + SEM. Two-tailed Student's *t*-test or two-way ANOVA followed by bonferroni post-test was used for statistical comparison. A p-value of <0.05 was considered to be statistically significant.

## Results

### Differential effects of sAβ and fAβ on miRNA regulation in mature primary cultured neurons

**Figure 1:**
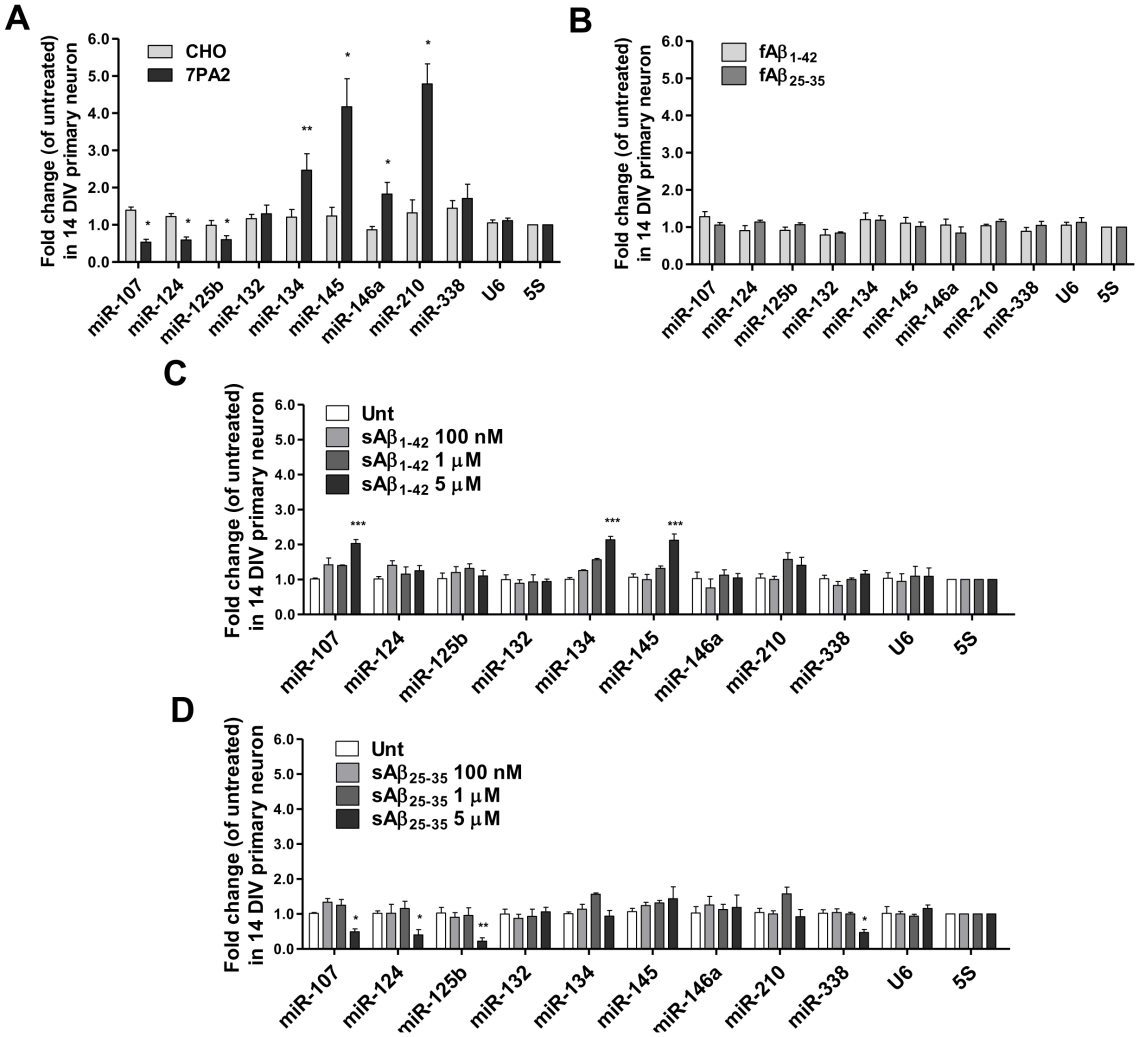
Differential regulation of miRNA expressions in primary cortical neurons by sAβ and fAβ. The 14 DIV rat primary neurons were either untreated or treated for 24(**A**) CHO or 7PA2 CM (n = 6); (**B**) 5 µM fAβ_25–35_ or fAβ_1–42_ (n = 4); **(C)** 100 nM, 1 µM or 5 µM synthetic sAβ_1–42_ (n = 3); **(D)** 100 nM, 1 µM or 5 µM synthetic sAβ_25–35_ (n = 3). qRT-PCR data were normalized to 5S rRNA. Two-tailed Student's *t*-test was used for statistical comparison for panels A and B. Two-way ANOVA followed by bonferroni post-test was used for panels C and D: *p<0.05, **p<0.01, ***p<0.001.

To investigate the regulatory effects of the two forms of Aβ (sAβ and fAβ) on neuronal miRNAs, we employed Aβ from two different sources: 7PA2 cell-secreted human sAβ and the synthetic sAβ and insoluble fAβ (human sequence). The 7PA2 cells (a CHO cell line containing stably overexpressed human APP_751_ with a V717F mutation) are known to secrete a mixture of Aβ monomers and toxic Aβ oligomers in the absence of insoluble aggregates [42]. A set of miRNAs whose target genes are involved in AD-related pathways were selected as readout. Table 1 summarized the known facts of the 9 miRNAs chosen for this study. We cultured neurons for 14 DIV until excitatory synapses were fully established. Based on our previous experience, the 7PA2 CM induced visible progressive dendritic damage to mature neurons from 3 to 24 hours but without massive cell death. We speculated that the degree of miRNA expression alteration must be positively associated with the severity of neuronal morphological damage. Therefore, we chose to first test in a 24-hour time period in order to screen miRNAs with potential roles in 7PA2 CM-triggered dendritic disruption and/or neuronal death. Mature neurons were treated with either 7PA2 CM or the control CHO CM, or simply left untreated for 24 hr. Strikingly, we found 7 of the 9 miRNAs levels were drastically altered by 7PA2 CM but not by CHO CM. Expressions of miR-107, miR-124 and miR-125b were suppressed by 40–60% (p<0.05, n = 6) upon 7PA2 CM challenge, as compared to those in CHO CM. Expressions of miR-134 (2.47-fold, p<0.01, n = 6), miR-145 (4.17-fold, p<0.05, n = 6), miR-146a (1.83-fold, p<0.05, n = 6) and miR-210 (4.79-fold, p<0.05, n = 6) were profoundly up-regulated by 7PA2 CM relative to CHO CM. MiR-132 and miR-338 displayed almost unaltered expressions in neurons (Fig. 1A).

Next, we examined the expression pattern of these miRNAs in fAβ-treated neurons. The fAβ were prepared from either Aβ_25–35_ or Aβ_1–42_. Successful fibrillation of Aβ_1–42_ was confirmed by SDS-PAGE and immunoblotting with a mouse monoclonal antibody B436 raised against human Aβ_1–12_ (a 6E10 equivalent) (Fig. S1A). Interestingly, the 9 miRNAs did not express differently after fAβ treatment for 24 hr; all changes were less than 2 folds and failed to reach statistical significance (Fig. 1B).

To validate our observation with 7PA2 CM, we tested the effect of synthetic soluble Aβ_1–42_ and Aβ_25–35_ peptides on these miRNA expressions. After oligomerization, the supernatant contained mainly Aβ monomers, dimers, trimers and tetramers (Fig. S1A). Consistently, we found that most of the 7PA2 CM-altered miRNAs were also regulated by either sAβ_1–42_ or sAβ_25–35_ (Fig. 1C and 1D). The results suggest that different Aβ species may have distinct preference for target miRNAs. The prepared monomeric Aβ_1–42_ species at a concentration of 5 µM did not cause significant change of the miRNAs in the treated neurons (Fig. S1B).

### Immunodepletion of Aβ from 7PA2 CM restored the selective neuronal miRNA expression altered by 7PA2 CM

**Figure 2:**
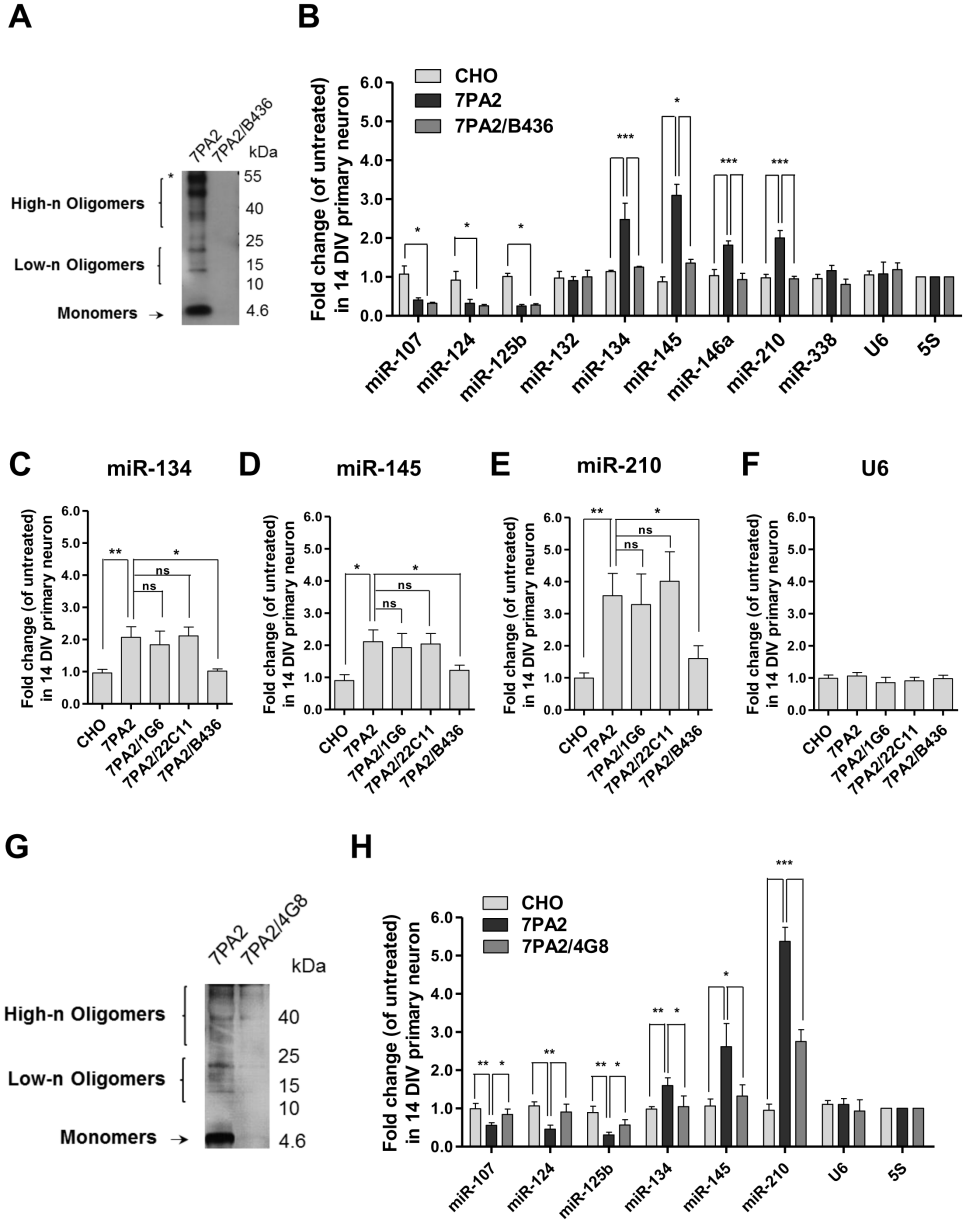
Immunodepletion of Aβ from 7PA2 CM restored the miRNA expressions altered by 7PA2 CM. Detection of sAβ species from 7PA2 CM before and after immunoprecipitation with B436 **(A)** or 4G8 **(G)**. In brief, 1 ml of 7PA2 CM was precipitated with TCA prior to SDS-PAGE, followed by Western probing with B436 or 4G8 as described in ‘Materials and Methods’. Asterisk indicates Aβ*56. **(B)** and **(H)** Neurons were untreated or treated with CHO CM, 7PA2 CM or 7PA2 CM immunoprecipitated with anti-Aβ antibody for 24 hr before being assayed for miRNA expression. **(C)** miR-134 **(D)** miR-145 **(E)** miR-210 **(F)** U6 snRNA expression levels in neurons untreated or treated with CHO CM, 7PA2 CM or 7PA2 CM immunodepleted with 1G6, 22C11 or B436 for 24 hr. (n = 3; two-tailed Student's *t*-test; *p<0.05, **p<0.01, ***p<0.001, ns stands for no significant difference).

To ascertain that the 7PA2 CM-altered neuronal miRNA expression was due to sAβ species, we first depleted the 7PA2 CM using B436. The specificity of this antibody to Aβ was determined by IPMS using 6E10 as a control (Fig S2A and S2B). After three rounds of immunoprecipitation, the 7PA2 CM, which was depleted of sAβ species as confirmed by immunoblotting (Fig 2A), was then added to the neurons for 24 hr. Strikingly, the sAβ-depleted 7PA2 CM almost completely restored the expression of miR-134, miR-145, miR-146a and miR-210, but not that of miR-107, miR-124 and miR-125b (Fig. 2B).

B436 reacts not only with N terminus Aβ, but also with soluble amyloid precursor protein α (sAPPα) and other APP fragments which contain the N terminal Aβ sequences existing in 7PA2 CM as recently reported [43]. To address the possibility of these N terminal APP fragments in the regulation of these miRNAs, we examined the effect of 7PA2 CM after immunodepleted with 1G6 that recognizes the APP epitopes N-terminally proximal to the beta-secretase 1 (BACE1) cleavage site, or with 22C11 that recognizes amino acid 66_–_81 of the N terminus on APP. We then selected 3 miRNAs whose levels were most dramatically altered (miR-134, miR-145 and miR-210) for subsequent assays. Intriguingly, as shown in Fig. 2C-F, the immunodepleted 7PA2 CM with either 1G6 or 22C11 was still able to alter the selected miRNA expressions. These findings suggest that the action of 7PA2 CM to change the selected miRNA expressions is independent of the N-terminal APP fragments.

We sought to establish whether the effects of 7PA2 CM on miR-107, miR-124 and miR-125b could be attributed to N-terminally truncated Aβ species. The 7PA2 CM was then immunodepleted with 4G8 (against amino acid 17–24 of Aβ) before being applied to neurons (Fig. 2G). Although there is technical limitation to confirm the removal of N-terminal truncated forms of Aβ from 7PA2 CM, the results clearly demonstrate a small but significant recovery of the three miRNAs (Fig. 2H). Therefore, we conclude that the 7PA2 CM-elicited modulation on selected miRNAs is mostly attributable to sAβ *per se*, but not other Aβ-sequence containing N-terminal APP fragments.

### Cell-derived sAβ induced time- and NMDAR-dependent alteration of miRNA expression

**Figure 3:**
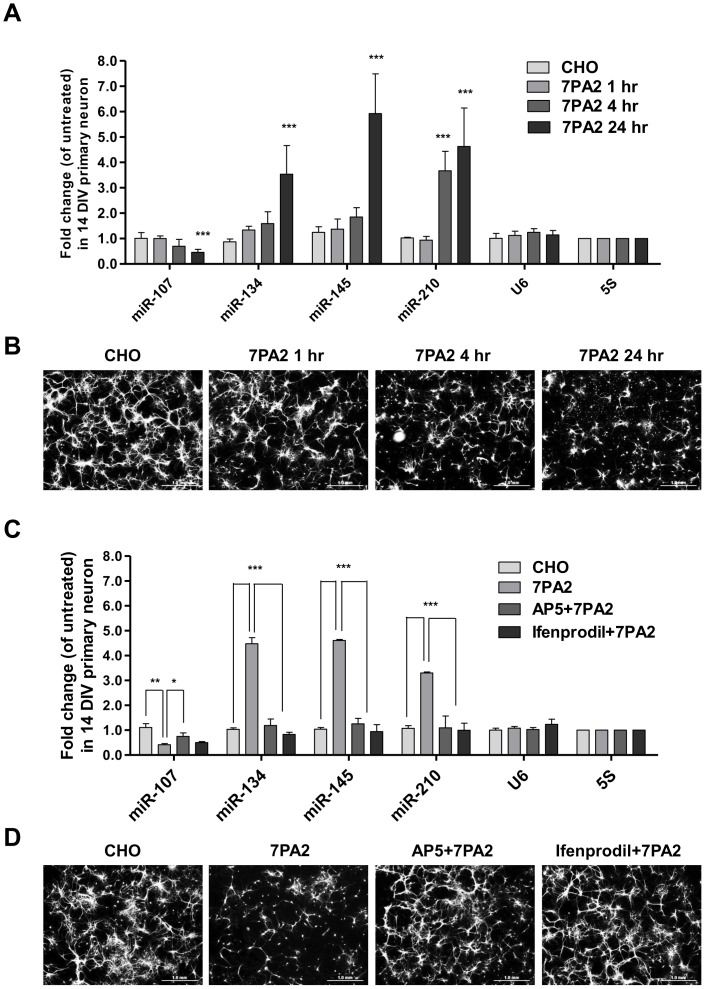
Cell-derived sAβ induced time- and NMDAR-dependent alteration of miRNA expression. **(A)** Temporal changes in miRNA levels after exposure to 7PA2 CM. Neurons were untreated, incubated with CHO CM for 24(n = 4; two-way ANOVA; ***p<0.001). **(C)** NMDAR antagonists restore 7PA2 CM induced alterations of miR-134, miR-145 and miR-210. Neurons were either untreated, treated with CHO or 7PA2 CM for 24 hr, or pretreated with either AP5 (50 µM) or Ifenprodil (10 µM) for 30 min before being incubated with 7PA2 CM. (n = 3; two-tailed Student's *t*-test; *p<0.05, **p<0.01, ***p<0.001). **(B)** and **(D)** Representative MAP2 immunostaining images of 14 DIV neurons.

We examined the temporal changes of the 4 miRNAs in neurons at 1, 4 and 24 hr after treatment with 7PA2 CM. MiR-107 was down-regulated by ∼50% at 24 hr (p<0.001, n = 4), while miR-134 and miR-145 were up-regulated 1.59- and 1.85-fold at 4 h (though failing to reach statistical significance) and 3.54- and 5.92-fold respectively at 24 hr (p<0.001, n = 4). MiR-210 was rapidly induced at 4 hr (3.67-fold, p<0.001, n = 4), and the effect sustained until 24 hr after treatment with 7PA2 CM (4.63-fold, p<0.001, n = 4). (Fig. 3A)

As reported in our recent study [44], exposure of rat primary neurons to 7PA2 CM caused rapid dendritic spine retraction, while prolonged exposure leads to synapse atrophy, dendritic breakage and eventually to neuronal death. The 7PA2 CM-induced miRNA alterations correlate with the timing of dendritic breakage as determined by MAP2 staining (Fig. 3B).

It has been proposed that sAβ exert their neurotoxicity through interaction with NMDAR via a postsynaptic site [45], [46]. The NMDARs are mainly non-synaptic in immature neurons before and during synapse formation (≦ 7 DIV), and are rapidly recruited to nascent synapses after synaptic contact or terminal differentiation (≧ 13 DIV) [47]. Therefore, the immature neurons under basal conditions normally lack of synaptic NMDARs. To probe the mechanism of sAβ-triggered deregulation of neuronal miRNAs, we tested the expression levels of the same set of miRNAs at 4 and 24 hr after 7PA2 CM challenge in younger neurons at 4 DIV, a time point when synaptic connections have yet to form. We found that only 3 out of the 9 miRNAs were significantly altered (*e.g.*, miR-107, miR-146a and miR-338, Fig. S3A). MAP2 staining of 4 DIV neurons indicates the absence of synaptic contact and sAβ-induced dendritic damage (Fig. S3B). This data suggest that the robust changes in the expression of the broader spectrum of miRNAs seen in mature neurons may be mediated through NMDAR.

To further test this hypothesis, we pre-incubated mature neurons with a non-selective NMDAR inhibitor, AP5 (50µM) or a selective NR2B receptor inhibitor, ifenprodil (10 µM) for 30 min before the application of 7PA2 CM. Interestingly, both AP5 and ifenprodil almost completely rescued not only the dendritic damage induced by sAβ (Fig. 3D), but also the disrupted expressions of miR-134, miR-145, and miR-210 (p<0.001, n = 3) (Fig. 3C), suggesting that activation of NR2B-containing NMDAR is required for the sAβ-mediated deregulation of these miRNAs in mature neurons. However, the reduction in miR-107 by 7PA2 CM was only partially corrected by AP5 (p<0.01, n = 3). Moreover, pretreatment with ifenprodil did not affect the 7PA2 CM-induced miR-107 suppression, suggesting that miR-107 down-regulation by 7PA2 CM does not act through NR2B-containing NMDAR and that an NMDAR-independent mechanism underlies this effect (Fig. 3C).

### Effect of sAβ in miRNA expression was not attributed to the attenuated insulin and PKA/CREB signaling

**Figure 4:**
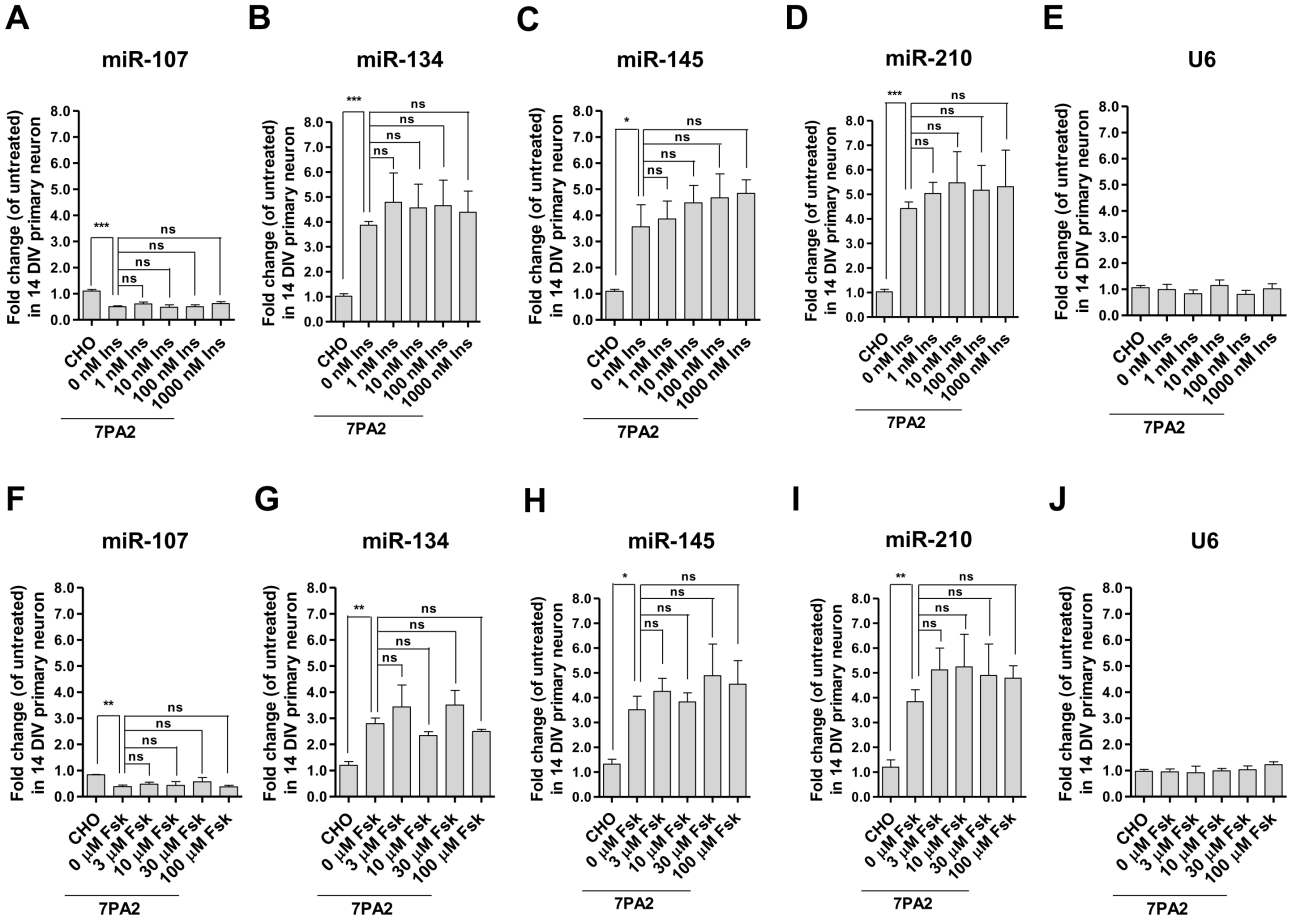
Effect of sAβ on miRNA expression was not attributed to the attenuated insulin and PKA/CREB signaling. Neurons were untreated, treated with CHO CM for 24(Ins) **(A-E)** or forskolin (Fsk) **(F-J)** for 30 min before exposure to 7PA2 CM for 24 hr. (n = 3; two-tailed Student's *t*-test; *p<0.05, **p<0.01, ***p<0.001, ns stands for no significant difference).

Aβ impairs memory likely in part through inactivating the PKA/CREB pathway or attenuating insulin signaling. Activation of these pathways has been demonstrated to be protective against Aβ toxicity [48], [49], [50]. We sought to test whether activation of the PKA/CREB or the insulin's neurotrophic signaling pathway can reverse the effect of sAβ-induced miRNA deregulation. We pretreated neurons with an inducer of cAMP, forskolin or human recombinant insulin at escalating doses for 30 min prior to the 7PA2 CM treatment. Insulin or forskolin dose-dependently respectively activated PI3K/AKT or PKA/CREB signaling in neurons within 15 min (Fig. S4A and S4B). We found that while neurons were protected against sAβ-elicited signaling impairment by insulin or forskolin (Fig. S4C and S4D), the miRNA expressional profiles in the treated neurons were not significantly different from those treated with 7PA2 CM alone (Fig. 4A-J). Hence, sAβ-mediated miRNA deregulation is likely not via inhibition of the CREB or insulin signaling.

### Oxidative stress served as the primary underlying mechanism to the repression of miR-107 by cell-derived sAβ

**Figure 5:**
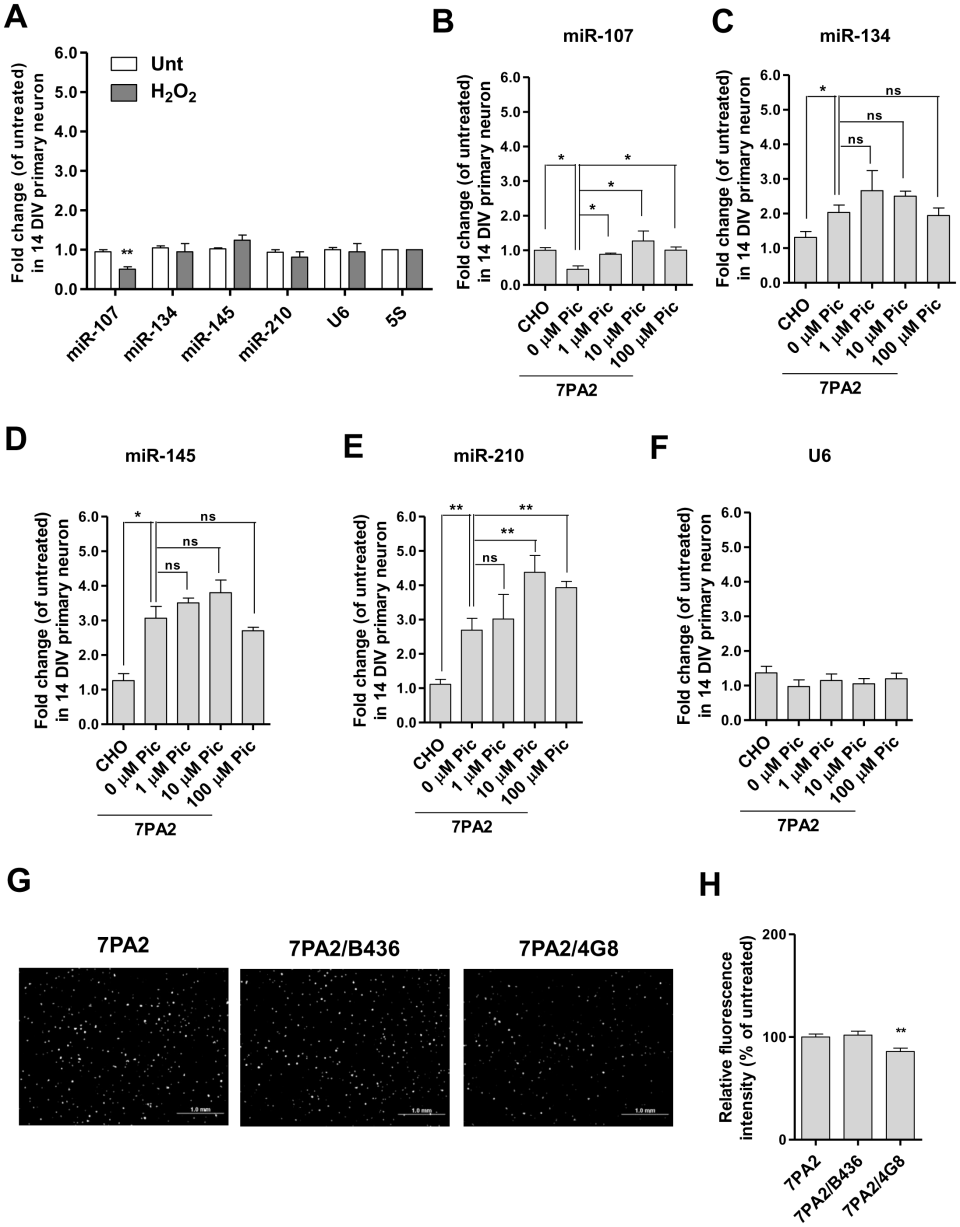
Oxidative stress served as the key trigger to down-regulate miR-107 by cell- derived sAβ. **(A)** Neurons were treated with or without 100 µM H_2_O_2_ for 4 hr. (n = 3; two-tailed Student's *t*-test; **p<0.01) **(B)** miR-107 **(C)** miR-134 **(D)** miR-145 **(E)** miR-210 **(F)** U6 snRNA expression levels in neurons with different treatments. Piceid (Pic) was added 30 min prior to the addition of 7PA2 CM. (n = 4; two-tailed Student's *t*-test; *p<0.05, **p<0.01, ns stands for no significant difference) **(G)** Representative intracellular ROS staining in neurons. **(H)** Quantification of ROS fluorescence intensity with Image J. (n = 3; at least 3 random fields per slide; two-tailed Student's *t*-test; compared to 7PA2 column; **p<0.01).

It has been reported that the neurotoxic effect of Aβ relies on the intracellular ROS production [51]. To probe whether Aβ-induced oxidative stress underlies miRNA alteration, we first measured the expressions of the selected miRNAs upon exposure to an exogenous H_2_O_2_ insult. The half-life of H_2_O_2_ in water ranges from 8 hr to 20 days. Based on our previous experience, H_2_O_2_ at a concentration ranging between 100–300 µM was sufficient to induce moderate neurotoxic effect but devoid of massive cell death. Herein, we chose to treat the neurons at 100 µM H_2_O_2_ for 4 hr prior to RNA isolation. Interestingly, only the miR-107 level was markedly reduced by H_2_O_2_ (∼50%, p<0.01, n = 3), suggesting that the suppression of miR-107 by sAβ may be mediated by an oxidative stress-elicited mechanism (Fig 5A). To test a direct involvement of ROS in 7PA2 CM-induced miRNA deregulation, we blunted the ROS signals in 7PA2 CM treated neurons by a strong antioxidant piceid. Piceid is a major derivative of resveratrol, but appears to be more efficacious in free radical scavenging [52] and (Liao unpublished data). Consistent with our hypothesis, piceid at as low as 1 µM rescued the repression of miR-107 by 7PA2 CM, but did not restore the expressions of the other three miRNAs even at higher concentrations (Fig. 5B–F), indicating that ROS is a contributor to 7PA2 CM-triggered down-regulation of miR-107. The conclusion is further supported by ROS staining showing that coincubation with 1 µM piceid was sufficient to attenuate the elevated ROS signals in 7PA2 CM treated neurons (Fig. S5A and S5B). Surprisingly, immunodepletion with none of the anti-N-terminal APP or Aβ antibodies (1G6, 22C11 or B436) could relieve the increase in ROS levels induced by 7PA2 CM (Fig. S5A and S5B), consistent with unaltered miR-107 expression (Fig. S5C). In addition, immunodepletion of 7PA2 CM with anti-mid-region Aβ antibody (4G8) yielded a subtle but significant decrease in ROS production (∼15%) in neurons as shown in Fig. 5G and 5H, consistent with the degree of recovery in miR-107 expression (Fig. 2H), indicating that the observed ROS elevation by 7PA2 CM may be in part be due to the N-terminal truncated Aβ species. Together, these data imply that N-terminally-truncated-Aβ-induced ROS production underlies the 7PA2 CM-triggered miR-107 suppression.

## Discussion

We produced three main findings here. First, we observed that the deregulations of certain miRNAs that have been previously identified in human AD brains could be reproduced in our primary neuronal model of rodent brains through treatment with sAβ from both natural and synthetic sources. Second, we found that a subset of miRNAs was robustly and selectively regulated by sAβ, but not fAβ. Third, our study revealed the impact of NMDAR signaling and ROS on sAβ-mediated miRNA deregulation. Despite the inherent imperfections in using rodent primary brain cells to study a process that affects the aged human brain, the perfectly conserved miRNA species identified in rodent primary neurons and human AD brains validates the sAβ-treated neuronal model we used. Most importantly, it adds to the growing body of supporting evidence that insults from sAβ species contribute to the cascade of events during AD pathogenesis. To our knowledge, this is the first report of selective deregulation of AD-relevant miRNAs induced by sAβ from a natural source, though there were previous studies that used aged fAβ [53].

Although we observed dysregulation on a similar set of miRNAs by 7PA2 CM and synthetic sAβ species, we noticed a drastic difference in the effective concentrations used. Moreover, the degrees of impact on miRNA expression induced by synthetic sAβ are not as large as that we observed using the cell-secreted sAβ_._ As measured by Aβ ELISA kit, the effective concentration of the Aβ species in 7PA2 CM is approximately 30 ng/mL (∼ 6.6 nM), which is close to the patho-physiological concentration of Aβ in CNS. In contrast, it requires at least 5 µM of the synthetic Aβ (effective concentration of which is approximately 250 nM [54]) to produce a similar degree of insults in neuronal morphology and miRNA alterations. For yet unknown reason, presumably owing to intrinsic thermodynamic instability of synthetic sAβ species, it has been frequently reported to use 1–5 µM synthetic sAβ to achieve neurotoxicity equivalent to a nanomolar range of sAβ from a natural source such as 7PA2 CM [55], [56].

It should be pointed out that the 7PA2 cell-derived sAβ species constitute not only low-n Aβ oligomers (*e.g*., dimers to tetramers), but also larger species of oligomers (*e.g.*, Aβ*56 decamer) (Fig. 2A and Ref [57]). A recent mass spectrometric characterization of the Aβ species in 7PA2 CM reveals that an array of proteolytic byproducts of APP and Aβ are presented [43], especially the N-termini which are similar to those found in human AD brains. Therefore, we cannot rule out the possibility that the most toxic Aβ*56 species or even some other soluble peptide species from 7PA2 can also modulate this set of miRNAs, which warrants further investigation. Nevertheless, the array of miRNAs dysregulated by sAβ as discovered in our cultured neurons may partially account for the cause of the pathologically altered miRNAs observed in AD brains to certain degrees.

In this study, we assessed sAβ-induced expressional changes in 17 neuronal miRNAs previously reported to have functions related to BACE1/APP regulation, oxidative phosphorylation, synaptic plasticity, apoptosis or inflammation. The 17 miRNAs are: miR-9, miR-29a, miR-29b-1, miR-34a, miR-101, miR-106b, miR-107, miR-124, miR-125b, miR-132, miR-134, miR-138, miR-145, miR-146a, miR-181b, miR-210, and miR-338. Those miRNAs whose levels were unaltered after a 24 hr exposure to either sAβ or fAβ were excluded from further study. We did not observe any changes in miR-34a and miR-106b, whose levels have been reported to be aberrant in transgenic mouse models for AD [36], [38]. These findings may reflect species-specific regulation of these miRNAs, as all primary neurons in this study were cultured from embryonic rats. Moreover, there was no noticeable change of expression in the APP-regulating miR-101 evoked by sAβ or fAβ. Though loss of miR-9, miR-29a and miR-29b-1 have been documented in sporadic AD brains, correlating with increased BACE1 protein expression [29], there is also conflicting evidence showing the opposite trend [27], [58], [59], [60]. In our preliminary study, we did not observe any significant changes to these miRNAs' expression by either Aβ forms, implying that any changes in expression could be independent of Aβ.

### MiR-107

Similar to the miR-29a/29b-1 cluster, miR-107 down-regulation has been observed in mild cognitive impairment (MCI), an early stage of AD; BACE1 has been shown to be a major miR-107 target site [28]. We show here that the level of miR-107 in mature neurons was markedly reduced by 7PA2 CM, partially reversed by AP5 or immunodepletion with an anti-mid-regional Aβ antibody (4G8). Interestingly, this 7PA2 CM-induced miR-107 reduction was not restored by immunodepletion with the anti-N-terminal APP fragments antibody (22C11 and 1G6) or an anti-Aβ_1-12_ antibody (B436). These results imply a potentially important role of the mid-regional truncated Aβ species in inducing ROS-like signals. Indeed, the mid Aβ fragment (*e.g.*, Aβ_25-35_) has been found to be more toxic than full-length Aβ in many studies. Further investigation revealed a similar degree of down-regulation of miR-107 upon H_2_O_2_ treatment. The reduction of the miR-107 levels by 7PA2 CM was completely rescued by an antioxidant piceid. Prior studies have reported that miR-107 is reactive to glucose concentration [61], [62], implying that multiple factors could be involved in miR-107 regulation such as elevated metabolic demands and/or oxidative stress in neurons during 7PA2 CM treatment. Based on the results from online search algorithms that predict miRNA targets, there are several AD-related gene targets other than BACE1 for miR-107, such as LRP1, CDK5, APP, BACE2 and Cofilin (Table 2). Therefore, dissecting how miR-107 is regulated in neurons is of particular importance in understanding its role in AD pathogenesis.

**Table 2:** List of selective putative or validated target genes for miR-107, miR-134, miR-145 and miR-210.

miRNA Name	Gene Targets Related to AD	Related Pathways to AD
miR-107	Lrp1 ○	APP processing; Aβ uptake
	Cdk5r1(p35) ○	Tau posttranscriptional modification
	App ○	Aβ generation
	Grn •	Glucose metabolism
	Bace1 •	APP processing
	Bace2 ○	APP processing
	Cfl1•	Dentdritic/synaptic dysfuction
miR-134	Pum2 •	Dendrite morphorgenesis; synaptic function; translational control
	Bdnf ○	Neuron survival; long-term memory
	Creb1 •	Transcriptional control; long-term memory formation
	Limk1 •	Brain development
	Limk2 ○	Brain development
miR-145	Grb10 ○	IGF-1/insulin signaling; neuroprotection; anti-apoptotosis
	Igf1r •	IGF-1/insulin signaling; neuroprotection; anti-apoptotosis
	Irs1 •	IGF-1/insulin signaling; neuroprotection; anti-apoptotosis
	Irs2 •	IGF-1/insulin signaling; neuroprotection; anti-apoptotosis
	Homer2 ○	Cell growth; inhibit Aβ production
miR-210	Iscu1/2 •	mitochondrial function
	Cox10 •	mitochondrial function
	Bdnf ○	Neuron survival; long-term memory
	Syngap1 ○	Axon formation; AMPA receptor trafficking; exitatory transmission
	Igf1r ○	IGF-1/insulin signaling; neuroprotection; anti-apoptotosis
		

Targets were predicted by TargetScan, PicTar, Microcosm, and EIMMo. ○, putative target genes; •, validated target genes.

### Inflammation

The up-regulation of miR-146a in the temporal cortices of AD patients has been consistently reported by several studies [58], [59], [63]. Its induction was shown to be dependent on NF-κB in response to IL-1β and Aβ_1–42_, or oxidative stress in cultured human neuronal glial cells [63], suggesting its involvement in inflammatory or oxidative stress pathways. In concordance, our study shows a selective up-regulation of miR-146a in both immature and mature neuron cultures by sAβ. However, it is difficult to distinguish between contributions from the neuronal and glial pools given that our primary culture contains both elements, with neurons predominating, as there are technical limitations in purifying neurons from embryonic rats.

### Synaptic plasticity

MiR-124, miR-125b, miR-132 and miR-134 are all abundantly expressed in the brain and regulate synaptic plasticity [19], [20], [21], [23]. Intriguingly, miR-134 not only can be induced by neuronal activity through the binding of MEF2 to its promoter region [17], but also has an inhibitory effect on spine development via Limk1 [19] and on memory via CREB [23]. Our study reveals that the increase of miR-134 is attributed to neuronal hyperactivity evoked by sAβ at the synaptic NMDA receptors. Given that miR-124 also has a role in CREB-targeting and constraining synaptic plasticity [20], its down-regulation by sAβ was surprising. Additionally, we expected sAβ to result in up-regulation of miR-125b and down-regulation of miR-132, as over-expression of miR-125b and miR-132 have opposite effects (reduced and enhanced, respectively) on synaptic strength [21]. However, our data here show rather a reduction in miR-125b and no change in miR-132 upon sAβ treatment. These unexpected results suggest a possibility of compensatory changes in miR-124 and miR-125b to boost synaptic strength.

Our finding of the robust induction of miR-145 and miR-210 is novel to the field. The majority of the information regarding these two miRNAs comes from cancer biology. Their functions in neurons will need to be carefully studied. In cancer, miR-145 appears to act as a tumor suppressor [64], [65] and its induction is thought to be dependent on p53 [66]. Enhanced p53 immunoreactivity has been associated with apoptosis in AD [67], [68]. Besides, p53 inhibition has been shown to protect neurons from amyloid-induced cell death [69]. It is highly plausible that the up-regulation of miR-145 is mediated via an Aβ-p53 pathway. Interestingly, the predicted and validated targets of miR-145 (Grb10, IGF-1R, IRS1 and IRS2) are convergent on IGF-1 signaling (Table 2), which is decreased in AD brains [70]. It is also of particular interest that in a recent report miR-145 was robustly up-regulated by fear conditioning [71], implying a potential role in learning and memory formation. MiR-210 is also viewed as a pro-apoptotic molecule increased under hypoxia condition via HIF-1α [72], [73]. Other than the validated gene targets ISCU1/2 and COX10, which have important roles in mitochondrial respiration and function, miR-210 is also predicted to target several neuroprotective proteins, such as BDNF, SYNGAP1 and IGF-1R. (Table 2)

**Figure 6:**
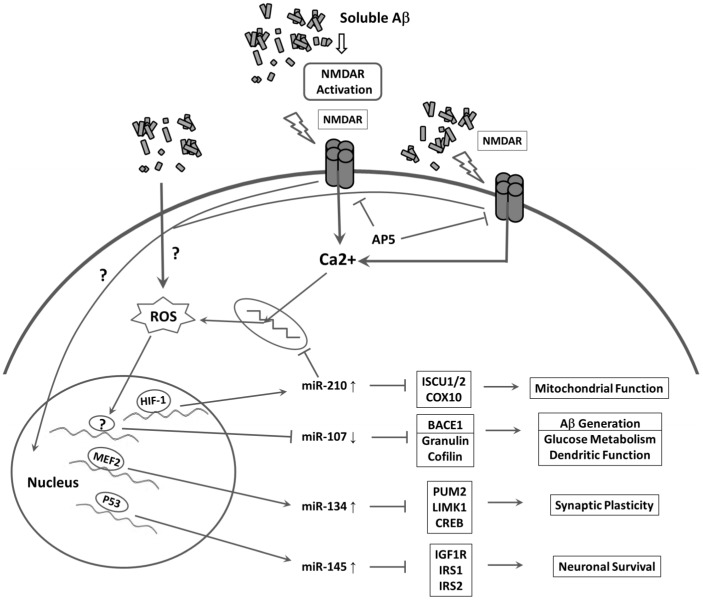
Schematic diagram of the identified sAβ-disrupted miRNA regulatory networks within a neuron. The sAβ leads to extrasynaptic NMDAR overactivation, excessive calcium influx, and subsequent increase in intracellular mitochondrion-derived ROS production. Alteration of miRNA levels in cell body follows transcriptional activation/repression of corresponding transcription factors in the nucleus, leading to AD-relevant target gene repression/activation and associated AD-type pathophysiological changes.

There are many hypotheses for AD pathogenesis, *e.g.* mitochondrial dysfunction, synaptic failure, apoptosis, DNA damage, nitrosative/oxidative stress, inflammation, insulin/IGF-1 resistance and lipid peroxidation; each receives considerable experimental supports. Our work adds further evidence for selective dysregulation of miRNAs-107, 134, 145 and 210 in primary neurons by sAβ species that may associate with or contribute to specific functional defects in ROS responses, synaptic plasticity and IR/IGF-1R signal transduction. Although we have not yet elucidated the underlying mechanism(s) of how these miRNAs are dysregulated by sAβ, our study sheds light on an NMDAR-dependent and/or oxidative stress-mediated mechanism (Fig. 6). We will further investigate how the expression levels of these miRNAs are altered. In particular, we will focus on addressing the following questions: validation of specific functions of those miRNAs that are altered at early time points (*e.g.*, miR-210 at 4 hr), the responsible transcriptional events as well as potential interplay between the up-regulated and down-regulated miRNAs. These studies will likely yield important information in terms of clarifying the specific roles played by miRNAs in AD pathogenesis.

## Supporting Information

Figure S1 **Monomeric Aβ did not alter the expressional level of selective miRNAs.** (**A**) Representative western blot showing mAβ_1-42_, soluble (sAβ_1-42_) and fibrillar (fAβ_1-42_) Aβ_1-42_. Lane 1: peptide prepared in HFIP/DMSO; Lane 2 and 3: peptide incubated at 4°C for 24 hr in PBS; Lane 4: peptide incubated at 37°C for 24 hr in PBS. **(B)** Neurons were treated with or without 5 µM synthetic mAβ_1-42_ for 24 hr. (n = 3; two-tailed Student's *t*-test).
(TIF)

Figure S2 **Detection of Aβ species by Mass Spectrometry.** Aβ peptides were immunoprecipitated as described in ‘Materials and Methods’ with either **(A)** 6E10 or **(B)** B436.
(TIF)

Figure S3 **Expressional profile of miRNAs in immature neurons treated with 7PA2 CM.** **(A)** Time-dependent expression of miRNAs upon exposure to 7PA2 CM in 4 DIV neurons. Neurons were treated for 4 and 24 hr. (n = 3; two-way ANOVA; **p<0.01, ***p<0.001). **(B)** Representative MAP2 immunostaining image.
(TIF)

Figure S4 **Insulin or forskolin protected neurons against sAβ-elicited signaling impairment.** **(A)** Dose-dependent activation of PI3K/AKT pathway by insulin. Neurons were treated with water or 1, 10, 100 or 1000 nM insulin for 15 min before being lysed. **(B)** Dose-dependent activation of PKA/CREB pathway by forskolin. Neurons were treated with DMSO or 3, 10, 30 or 100 µM forskolin for 15 min before being harvested. **(C)** Immunodepletion of 7PA2 CM with B436 or 4G8 restores 7PA2 CM attenuated activation of PI3K/AKT and CREB pathways. **(D)** Insulin or forskolin protects neurons against Aβ-impaired PI3K/AKT and PKA/CREB signaling. All treatments were performed for 24 hr. Insulin was treated at 1 µM. Forskolin was treated at 100 µM. Representative western blots and quantification of three independent experiments are shown (n = 3; two-tailed Student's *t*-test; *p<0.05, **p<0.01). Asterisks indicate non-specific bands.
(TIF)

Figure S5 **Piceid effectively blunted the ROS elevation induced by 7PA2 CM.** **(A)** Representative intracellular ROS staining in neurons with different treatments. **(B)** Quantification of ROS fluorescence intensity with Image J. (n = 3; at least 3 random fields per slide; two-tailed Student's *t*-test; compared to CHO column; *p<0.05, **p<0.01, ***p<0.001). **(C)** miR-107 expression levels in neurons with indicated treatments. (n = 3; two-tailed Student's *t*-test; *p<0.05, ns stands for no significant difference).
(TIF)
