# Supplementary figures and images for: Soluble Beta-Amyloid Peptides, but Not Insoluble Fibrils, Have Specific Effect on Neuronal MicroRNA Expression
Source: PLoS One. 2014 Mar 4;9(3):e90770. doi: 10.1371/journal.pone.0090770 (PMC3942478; doi:10.1371/journal.pone.0090770)

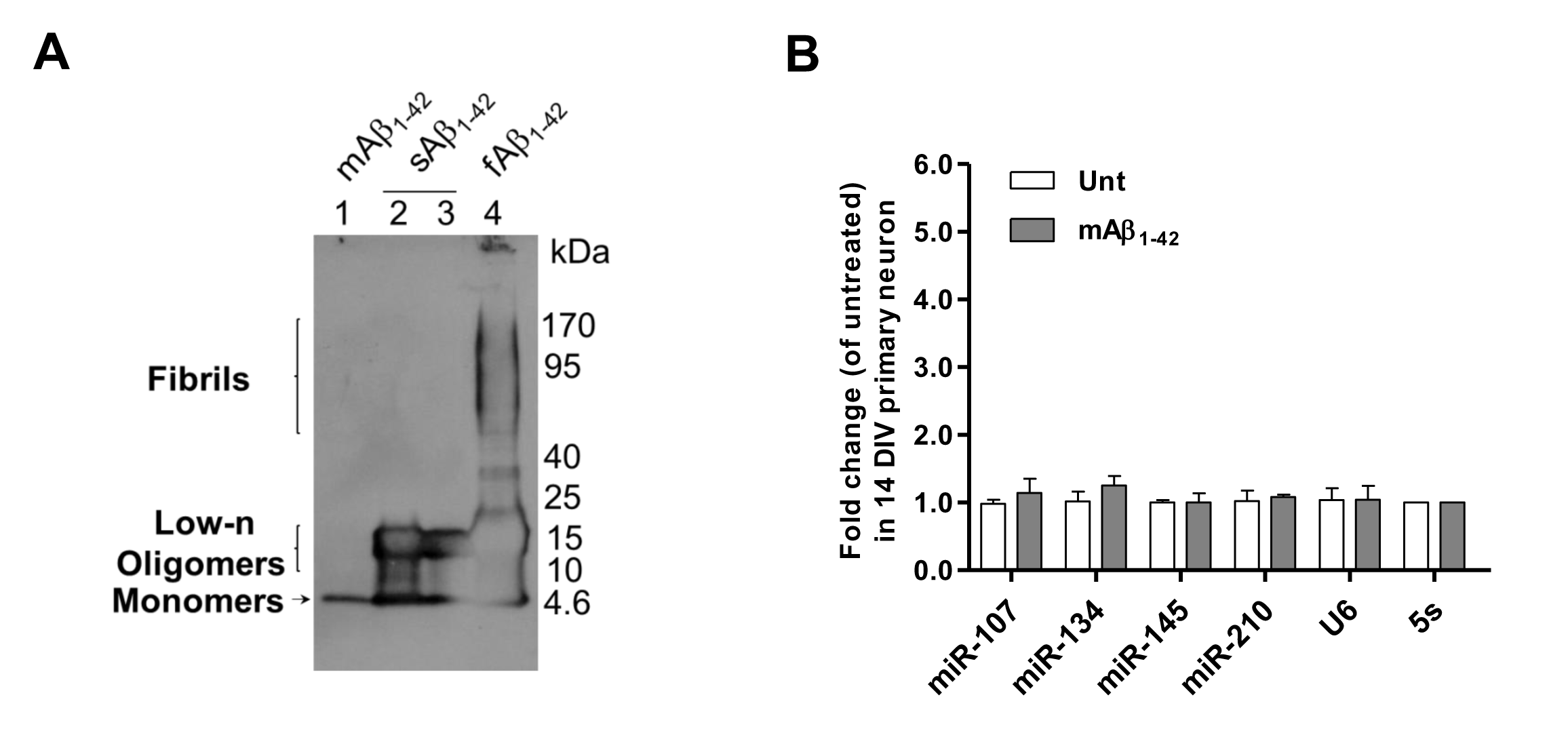

Supplement: Figure S1 — Monomeric Aβ did not alter the expressional level of selective miRNAs. (A) Representative western blot showing mAβ1-42, soluble (sAβ1-42) and fibrillar (fAβ1-42) Aβ1-42. Lane 1: peptide prepared in HFIP/DMSO; Lane 2 and 3: peptide incubated at 4°C for 24 hr in PBS; Lane 4: peptide incubated at 37°C for 24 hr in PBS. (B) Neurons were treated with or without 5 µM synthetic mAβ1-42 for 24 hr. (n = 3; two-tailed Student's t-test). (TIF) [file pone.0090770.s001.tif]

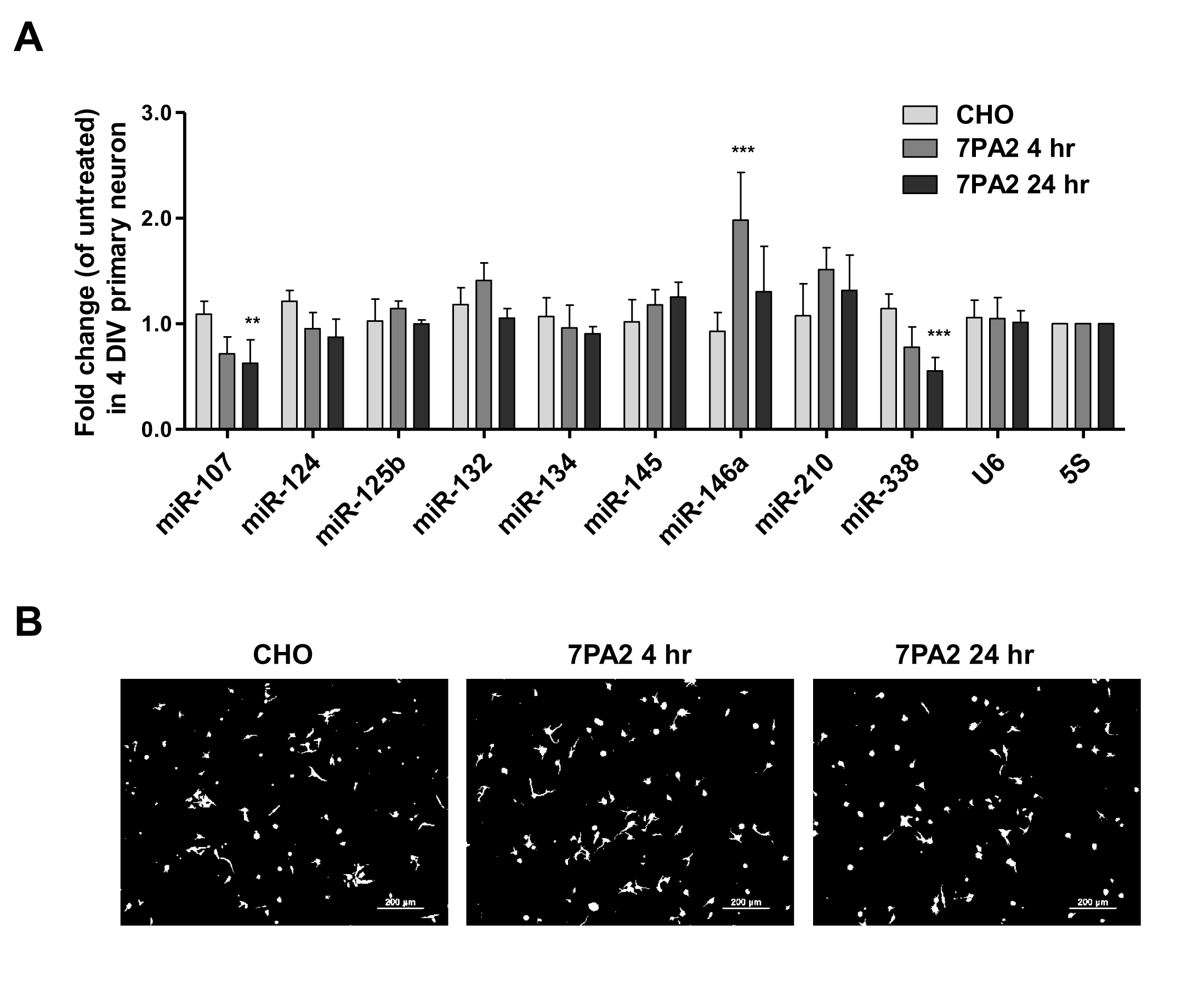

Supplement: Figure S3 — Expressional profile of miRNAs in immature neurons treated with 7PA2 CM. (A) Time-dependent expression of miRNAs upon exposure to 7PA2 CM in 4 DIV neurons. Neurons were treated for 4 and 24 hr. (n = 3; two-way ANOVA; **p<0.01, ***p<0.001). (B) Representative MAP2 immunostaining image. (TIF) [file pone.0090770.s003.tif]

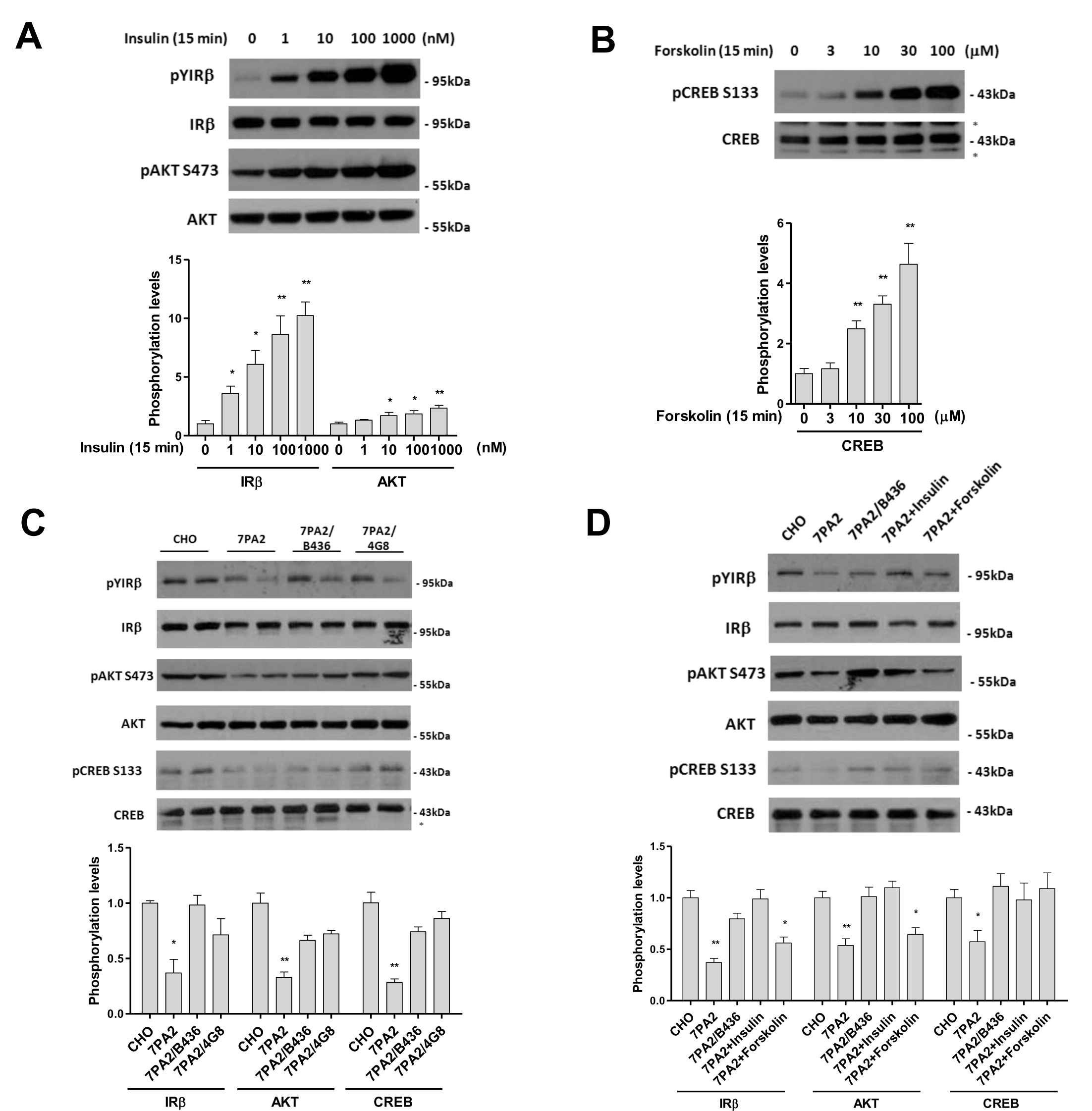

Supplement: Figure S4 — Insulin or forskolin protected neurons against sAβ-elicited signaling impairment. (A) Dose-dependent activation of PI3K/AKT pathway by insulin. Neurons were treated with water or 1, 10, 100 or 1000 nM insulin for 15 min before being lysed. (B) Dose-dependent activation of PKA/CREB pathway by forskolin. Neurons were treated with DMSO or 3, 10, 30 or 100 µM forskolin for 15 min before being harvested. (C) Immunodepletion of 7PA2 CM with B436 or 4G8 restores 7PA2 CM attenuated activation of PI3K/AKT and CREB pathways. (D) Insulin or forskolin protects neurons against Aβ-impaired PI3K/AKT and PKA/CREB signaling. All treatments were performed for 24 hr. Insulin was treated at 1 µM. Forskolin was treated at 100 µM. Representative western blots and quantification of three independent experiments are shown (n = 3; two-tailed Student's t-test; *p<0.05, **p<0.01). Asterisks indicate non-specific bands. (TIF) [file pone.0090770.s004.tif]

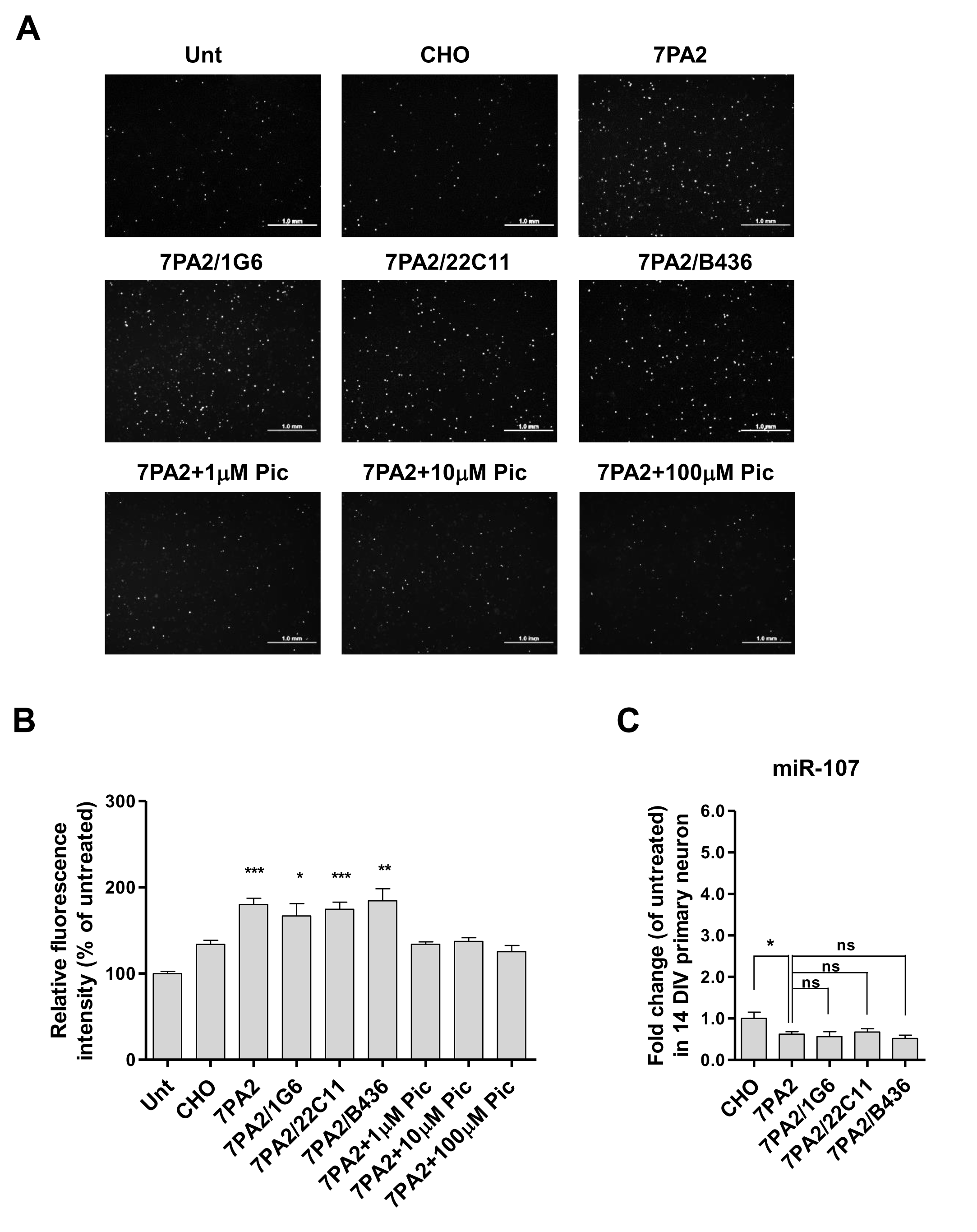

Supplement: Figure S5 — Piceid effectively blunted the ROS elevation induced by 7PA2 CM. (A) Representative intracellular ROS staining in neurons with different treatments. (B) Quantification of ROS fluorescence intensity with Image J. (n = 3; at least 3 random fields per slide; two-tailed Student's t-test; compared to CHO column; *p<0.05, **p<0.01, ***p<0.001). (C) miR-107 expression levels in neurons with indicated treatments. (n = 3; two-tailed Student's t-test; *p<0.05, ns stands for no significant difference). (TIF) [file pone.0090770.s005.tif]
